# Evaluation of the Effect of Mindfulness-Based Training on the Quality of Work-Life and Motivations of Nurses Working During the COVID-19 Pandemic

**DOI:** 10.5152/eurasianjmed.2023.23180

**Published:** 2023-10-01

**Authors:** Yasemin Erden, Nurgül Karakurt, Gülay İpek Çoban

**Affiliations:** 1 Department of Fundamental Nursing, Erzurum Technical University Faculty of Health Science, Erzurum, Turkey; 2 Department of Psychiatric Nursing, Erzurum Technical University Faculty of Health Science, Erzurum, Turkey; 3 Fundamentals of Nursing Department, Atatürk University Faculty of Nursing, Erzurum, Turkey

**Keywords:** Mindfulness, nurse, pandemic, work motivation, quality of work-life

## Abstract

**Objective:**

This study aims to determine the effect of mindfulness stress training given to nurses working during the COVID-19 pandemic period on the quality of work-life and motivation of nurses.

**Materials and Methods:**

The research was carried out as a pre-test and post-test control group quasi-experimental model from nurses working in a university hospital in eastern Turkey between September 2021 and December 2021. The study population consisted of 850 nurses working in the hospital. The sample consisted of 42 nurses (21 experimental, 21 control group) who agreed to participate in the study and met the inclusion criteria. In the study, selection bias was controlled by randomized assignment and concealing randomization. The nurses’ names were grouped alphabetically and randomized using the research randomizer program. Within the scope of the research, the mindfulness Stress Training Program was applied to the nurses in the experimental group. Data were collected using the Personal Information Form, Nurse Work-Life Quality Scale, and Nurse Work Motivation Scale. Chi-square test, Mann–Whitney *U* test, and Wilcoxon marking tests were used to analyze the data.

**Results:**

It was determined that the total score of the work motivation scale of the nurses in the experimental group was 48.42 ± 5.39 before the training, 59.52 ± 6.52 after the training, and the total score of the nurse work-life quality scale was 81.00 ± 12.46 before the intervention and 91.08 ± 11.06 after the intervention. The post-test scores of the control and experimental groups were statistically significant (*P* < .05).

**Conclusion:**

It was found that the Mindfulness-Based Stress Reduction program was effective in nurses’ work motivation and quality of work-life during the pandemic period.

**Main Points**

- The mindfulness-based stress training given was affective on the quality of life and job motivations of nurses.
- Providing face-to-face training to nurses during epidemic periods such as pandemics will provide very positive results.
- Increasing the work motivation and quality of life of nurses will also increase the quality of health care.

## Introduction

The COVID-19 virus, which emerged in Wuhan, China, in December 2019 and spread worldwide in a short time was declared a pandemic by the World Health Organization; it continues to affect the whole world through various mutations.^1^ The prevalence of COVID-19 in the general population in many countries, its novel, unpredictable, and highly infectious nature, the requirement of physical distance and isolation, and associated high rates of morbidity and mortality make the usual forms of coping dysfunctional, and require the development of new ways of adaptation to the crisis as well as making plans about the crisis. It places an unprecedented burden on all healthcare professionals worldwide.^2,3^ In addition to the increase in workload, nurses are more focused on providing end-of-life care by having to adapt to the new normals.^3^ On the other hand, due to the increasing number of patients, the working hours of the nurses have changed, and they have had to work for a long time. Nurses from different fields have been assigned to meet the need for nurses. They were working in these unfamiliar areas, such as intensive care, which require knowledge and skills, further increasing the work stress of nurses working both in their newly assigned duties and in the clinic. Interaction between nurse and patient was limited with the addition of protective measures such as mask-glasses-visor and the factors mentioned earlier.^4^ These changes in the pandemic lead to psychosocial problems in nurses. As a result of many studies, the pandemic has been found to lead to mental problems such as post-traumatic stress disorder, anxiety, depression, sleep disorder, and despair in nurses.^5-7^ All these negative emotions affect nurses’ work-life and job motivation. Quality of work-life is generally defined as strengths and weaknesses in the work environment. At the same time, it is an objective and subjective concept that covers not only the physical but also mental, psychological, and social needs of the person.^8^ The low quality of work-life of nurses during the COVID-19 pandemic can also affect the care given to patients and cause negativity in the recovery process. In this context, nurses will be able to accept negative and positive emotions during the ongoing pandemic instead of destroying negative and undesirable emotions with the help of mindfulness-based emotional regulation programs.

The concept of mindfulness has increasingly attracted the attention of scientific research literature in recent years.^9,10^ Mindfulness is defined as accepting and approving of the moment without being influenced by the past or potential experiences and emotions.^11^ In other words, it is a mind and body practice that involves focusing attention on instantaneous experiences and observing inner experiences.^9,12^ The mindfulness-based therapies use various techniques and exercises that raise the level of awareness. With the implementation of this method, one learns strategies for dealing with problems, accepting internal and external experiences, and moving away.^13^

The mindfulness-based practices have been proven to have a positive effect on depression and anxiety.^14,15^ The mindfulness-based psychotherapy applications have also been widely used in the health field. Studies have shown that decreased anxiety, behavioral problems, and symptoms of depression were effective in coping with stress.^16,17^

Based on the studies, it can be stated that mindfulness-based therapies positively affect psychological well-being. As a result of the information obtained in this context, the study aims to determine whether the mindfulness-based cognitive therapy program during the pandemic impacts nurses’ quality of work-life and job motivation.

## Materials and Methods

### Research Design

This study is experimental research with a randomized controlled pre-test, post-test, and control group.

### The Location and Time of the Research

The study was conducted between September 2021 and December 2021 at a university hospital in eastern Turkey. The study population consisted of 850 nurses working in the university hospital (n = 850).

A priori power analysis was performed to determine the research’s sample size. In the power analysis, the inclusion of at least 20 nurses (10 experiments and 10 controls) with 95% power and 95% confidence levels was calculated using the G-power program to have a significant unit effect size. Based on the sample size determined for the main variable of the study, the sample size was determined with 52% backup as 42 nurses (21 experiments, 21 controls).^18^ Necessary announcements were made through the nurse in charge of the education unit of the hospital where the study will be conducted. After obtaining the necessary permissions, each nurse, who met the research inclusion criteria, was contacted face-to-face and informed about the purpose and implementation of the research. Their consent was obtained for inclusion in the study.

In the study, selection bias was controlled by randomized assignment and concealing randomization. The nurses’ names were grouped alphabetically and randomized using the research randomizer program. The individuals in the experimental and control groups were hidden from the researcher until starting the interventions.

### Research Inclusion Criteria

1. Working as a nurse at the AnUniversity Hospital
2. Having filled out the informed consent form in writing/verbally to participate in the study.
3. Not having participated in mindfulness-based training before.

### Research Exclusion Criteria

1. The emergence of a physical or mental problem in individuals during the research.
2. Individuals who want to leave the research when responding to the survey questions.
3. Having participated in mindfulness-based training before.

### Criteria for Removing from the Research

1. Not participating in any sessions of the training program.
2. Not participating in any of the study’s data collection stages (pre-test or post-test).

### Variables of the Study

#### Independent Variables:

- Mindfulness-based training

#### Dependent Variables:

- Quality of Nursing Work-Life Questionnaire (QNWLQ)
- Nurses Job Motivation Scale (NJMS)

### Research Hypotheses

**H** **_0_** = Mindfulness-based training does not positively affect the score averages of the participants in the QNWLQ and NJMS.

**H** **_1_** = Mindfulness-based training positively affects the score averages of the participants in the QNWLQ and NJMS.

### Instruments

In the collection of data, the Descriptive Characteristics Form, QNWLQ, and NJMS were used.

### Descriptive Characteristics Form

The researchers prepared this form which consisted of 12 items to identify the descriptive characteristics of nurses such as age, gender, marital status and work environment characteristics such as the type of their work and the stress they experience in the work environment.

### Quality of Nursing Work-Life Questionnaire

Developed by Brooks^19^ (2001), the scale was adapted into Turkish, together with its validity and reliability study, by Sirin and Sokmen.^20^ Quality of Nursing Work-Life Questionnaire consists of 35 items in 5 subscales: work environment, relations with managers, work conditions, work perception, and support services. The scale is a 5-point Likert-type scale; the 3rd, 11th, 16th, and 20th items are reverse coded, and the increase in the total score on the scale indicates that the nurses’ quality of work-life is high, and a decrease in the total score indicates poor quality of work-life. The Cronbach’s alpha value of the original scale was 0.89, and the Cronbach’s alpha value in this study was calculated as 0.86.

### Nurses Job Motivation Scale

The scale developed by Engin and Cam (2016)^21^ to determine the job motivation levels of nurses is a 3-point Likert-type scale (1 = disagree, 2 = partially Agree, 3 = agree). The 25-item questionnaire has no subscales. Cronbach’s alpha internal consistency coefficient of the questionnaire, α, was calculated as 0.847. The lowest and highest scores on the scale are 25 and 75, respectively. These data show that high scores on the scale indicate higher job motivation. As a result of the evaluations made on the NJMS, the validity and reliability levels were found appropriate for the nurse profile.^21^ Cronbach’s alpha value of this study was 0.83.

### Data Collection

Regarding the data collection and evaluation:

- The necessary permissions were obtained before starting the research.
- Pre-test measurements were made by face-to-face interviews with volunteer nurses in the experimental and control group (who meet the inclusion criteria) and informed them about the research.
- Nurses were divided into experimental and control groups by simple randomization principles.
- In the single-centered research, the first measurements of the control group were made to prevent interaction between the experimental and control groups. Then the experiment group’s measurements were made.
- It was decided with the group which days and between which hours the applications would be carried out with the experimental group so as not to disrupt the institution’s functioning and not to impose an extra workload on the nurses.
- In line with the decided plan, the experimental group was given Mindfulness-Based Stress Reduction (MBSR) training, consisting of 8 sessions of 40-50 minutes each, for 8 weeks and 1 session per week.
- In the sessions, the first 15-20 minutes were planned as a presentation about mindfulness, and the remaining 25-30 minutes were planned and implemented to share meditation practices and experiences. Each session focused on different issues related to mindfulness (concept of awareness, breathing exercise, body scan, autopilot concept, exploring nature, etc.).
- The MBSR program originated from 2500 years of meditation and was developed by Jon Kabat Zinn in the 1970s based on cognitive behavioral therapy, among the third-wave therapies. In this study, the 8-week MBSR program was applied to nurses together with the application of a semi-structured program. In this context, the program content was planned by determining the gains to achieve the goal.
- By determining the program’s needs, gains were planned in line with the data collected by reviewing national and international studies.
- Each session was planned in line with the targeted gains, the targets achieved (if not, why) were noted by the researcher at the end of each session, and the next session was planned accordingly.
- PowerPoint, subject-oriented video screenings, and interactive narration techniques were used for the presentation.
- The researchers were given assignments based on the themes of each week (body scan, breathing exercise, mindful eating, etc.). They were asked to receive feedback on these assignments before and after application. Feedback from each participant was obtained at the end of the sessions.
- The researcher has received the necessary training and certificate to provide mindfulness-based training. After completing all the training, the questionnaires were handed out to the nurses in the experimental group to fill out.
- In line with the equality principle of the research, the MBSR program was applied to the nurses in the control group after completing the training program of the experimental group and carrying out their post-tests.

### Data Analysis

Analyses were performed using Statistical Package for Social Sciences Statistics version 22.0 (IBM Corp., Armonk, NY, USA). Descriptive tests (percentage, arithmetic mean, standard deviation, min-max values) were used in the analysis of individual characteristics, the chi-square test was used to compare descriptive features between groups, the Mann–Whitney *U*-test was used to compare scale score averages between groups, and the Wilcoxon rank-sum test was used to compare intra-group scale score averages.

### Challenges and Limitations of the Study

The research is limited to the nurses in the An University. Since it was carried out in a single center and on the specified dates, the results can only be generalized to this study group.

### Ethical Approval

To carry out the study, ethical approval was obtained from the Atatürk University Faculty of Medicine Clinical Research Ethics Committee (Approval no: B.30. 2.ATA .0.01 .00/5 32, Date: December 11, 2020). Verbal consent was obtained from the nurses who participated in the study.

## Results

In the study evaluating the effect of mindfulness training on nurses’ quality of work-life and job motivation, the average age of the nurses in the training group was 36.42 ± 3.90, and the working years average was 15.8 ± 4.76. Of the nurses, 52.4% were married, 57.1% did not have children, 52.4% were cooperating with colleagues, 52.4% were satisfied with their job, 52.4% were working in shifts, 61.9% had a chronic disease, 76.2% did not exercise regularly, 76.2% described herself as pessimistic, and 33.3% gave 10 points to stress levels in the work environment.

The mean age of the nurses in the control group was 36.33 ± 3.82, and the mean working time was 16.00 ± 5.21. Of these nurses, 52.4% were married, 52.4% had children, 61.9% were not cooperating with colleagues, 61.9% were not satisfied with their job, 52.4% were working only in the day shifts, 71.4% did not have a chronic disease, 85.7% did not exercise regularly, 76.2% described herself as pessimistic, and 28.6% gave 10 points to stress levels in the work environment.

The nurses in the training and control groups were found to be similar in terms of age (*t* = −0.114, *P* = .910), working time (*t* = −0.038, *P* = .970), marital status (*U* = 220.50, *P* = 1.00), status of having children (*U* = 210.00, *P* = .759), cooperation with colleagues (*U* = 199.50, *P* = .538), job satisfaction (*U* = 199.50, *P* = .538), type of work (*U* = 210.0, *P* = .760), chronic disease status (*U* = 199.5, *P* = .538), regular sports status (*U* = 199.5, *P* = .437), self-identification status (KW = 0.197, *P* = .657), points given to the stress level in the work environment (KW = 0.285, *P* = .593) (*P* >.05) (Table 1).

In Table 2, according to the comparison of the NJMS pre-and post-test score averages of the nurses in the training and control groups, the pre-test NJMS average score of the nurses in the training group was 48.42 ± 5.39, and their post-test score average was 59.52 ± 6.52. The pre-test NJMS average score of the nurses in the control group was 48.85 ± 5.63, and their post-test score average was 47.66 ± 4.49.

After the training, the average post-test score of the nurses in the training group was found to increase compared to their pre-test score average, and the difference between the 2 scores was statistically significant (*P* < .001). The average post-test score of the nurses in the control group was found to decrease compared to the pre-test score average, but the difference was not statistically significant (*P* > .05).

It was found that the pre-test score average of the nurses in the training group was close to the average score of the nurses in the control group, and this difference was not statistically significant (*P* > .05). It was found that the difference between the post-test score averages of the nurses in the training and control groups was statistically significant (*P* < .001).

In Table 3, according to the comparison of the QNWLQ pre-and post-test score averages of the nurses in the training and control groups, the pre-test QNWLQ average score of the nurses in the training group was 81.00 ± 12.46, and their post-test score average was 91.08 ± 11.06. The pre-test NJMS average score of the nurses in the control group was 82.57 ± 13.91, and their post-test score average was 79.00 ± 13.16.

After the training, the average post-test score of the nurses in the training group was found to increase compared to their pre-test score average, and the difference between the 2 scores was statistically significant (*P* < .001). The average post-test score of the nurses in the control group was found to decrease compared to the pre-test score average, but the difference was not statistically significant (*P* > .05).

## Discussion

The study’s findings, which were carried out to evaluate the effect of mindfulness-based training given to nurses working during the COVID-19 pandemic on their quality of work-life and job motivations, were discussed with the relevant literature.

It was found that the nurses in the study were female, their average age was 36.42 ± 3.90, the majority were married, had children, had an average working time of 15 years, and the majority were working in shifts. In addition, most nurses have stated that they have cooperation with their colleagues and are satisfied with their work. In addition, it was found that most nurses have a chronic disease, do not exercise regularly, describe themselves as pessimistic, and a significant proportion of nurses rate 10 points the level of stress in the work environment (Table 1).

Today, individuals may face stressful situations (stressors) in many areas of life. Stress caused by stressors encountered in the working environment is called work stress.^22^

The work stress that occurs in work-life is related to many concepts such as the employees’ health, quality of life, job motivations, and work efficiency.^23^

According to the comparison of the NJMS pre-and post-test score averages of the nurses in the training and control groups in Table 2, the pre-test score average of the nurses in the training group was found to be 48.42 ± 5.39. The nurses’ average pre-test NJMS score in the control group was 48.85 ± 5.63, and the difference between them was not statistically significant (*P* > .001).

Considering that the minimum and maximum scores to be taken from the scale are 25 and 75, respectively, and that the job motivation is high in those with high scores, it can be stated that the pre-test nurse job motivation scale scores of the individuals in the training and control groups were below the average.

Looking at the studies in the literature with similar findings, it was found that the job motivations of the nurses were moderate level or below average^24-28^

The necessity to perform their jobs by putting human life first, and the fact that the main subject of their work is human life, leads to a high level of stress in health care professionals. The motivation levels of the personnel working under intense stress are expected to decrease. Low motivation is very important for the health institution as well as one’s life energy. Loss of motivation in the employee can also be a factor that compromises patient safety.^29^

According to Table 2, in the comparison of the NJMS pre-and post-test score averages of the nurses in the training and control groups, the post-test score average of the nurses in the training group was found to be above the average score of the scale (59.52 ± 6.52), whereas the average score of the nurses in the control group was below the average (47.66 ± 4.49), and the difference between them was statistically significant (*P* < .001).

According to the comparison of the NJMS pre-and post-test score averages of the nurses in the training and control groups in Table 2, the pre-test score average (48.42 ± 5.39) of the nurses in the training group was below the average of the scale, while their post-test score average (59.52 ± 6.52) was above the average of the scale. The difference was statistically significant (*P* < .001). While the NJMS pre-test score average of the nurses in the control group was below the average score of the scale (48.85 ± 5.63), it was found that the post-test score average was below the pre-test score average (47.66 ± 4.49), and the difference between them was statistically significant (*P* < .05).

Looking at the results, the NJMS post-test score average of the nurses in the training group was above the average score of the scale, indicating that the mindfulness training provided to nurses can be effective.

Mindfulness involves recognizing what is happening in the present moment and welcoming all the things that have been noticed. Therefore, mindfulness can be called a method of awareness consisting of a combination of perception and acceptance of this perception. Perception alone is just attention. Mindfulness is to perceive the present moment with a conscious mind and an open, loving, and compassionate heart.^9^

In a study in which Horner et al^30^ assessed the effect of the mindfulness training program on nurses, the post-test score averages of the control group remained the same, while the mindfulness scale score averages of the training group increased. It is also emphasized in the study that burnout and stress scores decrease with this increase. Gauthier et al^31^ on the other hand applied a MBSR program to nurses. After this application, the average mindfulness score of nurses increased. In the same study, it was found that stress and emotional fatigue scores decrease in line with the increase in mindfulness score.^31^ In this study, the increase in job motivation after mindfulness-based training was found to be compatible with the literature.

According to the comparison of the QNWLQ pre- and post-test score averages of the nurses in the training and control groups in Table 3, the pre-test score average of the nurses in the training group was 81.00 ± 12.46, the average score of the nurses in the control group was 82.57 ± 13.91, and the difference between them was not statistically significant (*P* > .001).

Considering that the lowest score to be taken from the scale is 35 and the highest score is 175, the increase in the total score indicates that the nurses have a high quality of work-life, and the decrease in the total score indicates that poor quality of work-life, it can be stated that the nurse job motivation scale pre-test score averages of the individuals in the training and control group were below the average.

In the studies conducted using the QNWLQ, in addition to studies that found a moderate level quality of work-life score in nurses,^20,32,33^ one study on the quality of work-life and affecting factors found that a third of nurses rated their quality of work-life as poor, and the majority of these nurses was found to plan to work in the hospital for less than a year or at most one year.^34^

Overtime work and work overload directly affect the lives of employees outside of work. The balance between the employee’s work and life outside work has an impact on the quality of work-life. The excess workload leads to a decrease in the quality of social life, less interaction with many people such as family and friends, as well as severe self-sacrifice in basic needs such as sleep, self-care, eating, and drinking. These sacrifices can lead to more chronic fatigue, more stress, and stress-related diseases, inadequacy in the role of the individual and family health problems, decreased quality of service, and significant social problems, especially in health care.^34^

According to Table 3, in the comparison of the QNWLQ pre- and post-test score averages of the nurses in the training and control groups, the post-test score average of the nurses in the training group (91.08 ± 11.06) was close to the average of the scale. The NJMS post-test score average of the nurses in the control group was below the average score that can be taken from the scale (79.00 ± 13.16), and the difference between them was statistically significant (*P* < .05).

According to the comparison of the QNWLQ pre- and post-test score averages of the nurses in the training and control groups in Table 3, the pre-test score average (81.00 ± 12.46) of the nurses in the training group was below the average of the scale, while their post-test score average (91.08 ± 11.06) was close to the average of the scale, and the difference was statistically significant (*P* < .001). While the NJMS pre-test score average of the nurses in the control group was below the average score of the scale (82.57 ± 13.91), it was found that the post-test score average was below the pre-test score average (79.00 ± 13.16), and the difference between them was not statistically significant (*P* > .05).

Looking at the results, the QNWLQ post-test score average of the nurses in the training group was above the pre-test score average, close to the average score that can be taken in the questionnaire, indicating that the mindfulness training provided to nurses can be effective.

Health is defined as a biopsychosocial optimal state of well-being, which is compatible with all elements of working life and allows employees to motivate themselves and their environment. Based on this definition, work-life and working conditions can directly affect the health of employees.^35,36^

Declining job satisfaction and happiness are important factors in disrupting the professional healthcare environment, affecting the job satisfaction of employees, as well as negatively affecting the patient care and well-being of the healthcare professionals.^37,38^

The concept of mindfulness, which allows people to perceive things as they are and to realize the present facts, is becoming increasingly popular today. It is also noteworthy that there has been a significant increase in the number of studies conducted in the field, especially in recent years. Studies have shown that the inclusion of mindfulness in daily life can make a very positive contribution to one’s life. It has been shown to be especially useful in groups that work under high stress.^39-42^ In this regard, the results of the study are in line with the literature.

As a result, it was found that the quality of work-life and job motivation scale scores of the nurses working during the COVID-19 pandemic were below average and that the mindfulness-based stress training given was effective on the quality of work-life and job motivations of nurses. It is believed that the face-to-face MBSR training program given to nurses, working intensively during the pandemic, will contribute significantly to the literature in terms of the results obtained. In this context, it is recommended that nurses improve themselves about MBSR, participate in certificate programs, assume an active role in the implementation of MBSR, and repeat the study with different sample groups.

## Figures and Tables

**Table 1.** Descriptive Properties of Groups (n = 42)

Characteristics	Education (n = 21)		Control (n = 21)		*t*	*P*
	Mean ± SD		Mean ± SD			
**Age**	36.42 ± 3.90		36.33 ± 3.82		−0.114	.910
**Year of study**	15.8 ± 4.76		16.00 ± 5.21		−0.038	.970
	**S**	**%**	**S**	**%**	U	*P*
**Marital status**						
Married	11	52.4	11	52.4	220.50	1.000
Single	10	47.6	10	47.6		
**Status of having a child**						
Yes	9	42.9	11	52.4	210.00	.759
No	12	57.1	10	47.6		
**Do you cooperate with your colleagues?**						
Yes	10	47.6	8	38.1	199.50	.538
No	11	52.4	13	61.9		
**Are you satisfied with your job?**						
Yes	10	47.6	8	38.1	199.50	.538
No	11	52.4	13	61.9		
**How do you work?**						
Daytime	10	47.6	11	52.4	210.00	.760
Night	11	52.4	10	47.6		
**Do you have any chronic diseases?**						
Yes	8	38.1	6	28.6	199.50	.518
No	13	61.9	15	71.4		
**Do you exercise regularly?**						
Yes	5	23.8	3	14.3	199.50	.437
No	16	76.2	18	85.7		
**How would you describe yourself?**					**KW**	***P***
Happy	6	23.8	4	19.0	0.197	.657
Witty	0	0	1	4.8		
Pessimistic	15	76.2	16	76.2		
Other	0	0	0	0		
**What is your score for the stress level in the work environment?**						
7	4	19.0	4	19.0	0.285	.593
8	5	23.8	5	23.8		
9	5	23.8	5	23.8		
10	7	33.3	7	33.3		

**Table 2.** Intra-Group and Inter-Group Comparison of Nurses Job Motivation Scale Pre-Test-Post-Test Mean Scores of Training and Control Group Nurses (n = 42)

	Education (n = 21)	Control (n = 21)	Test and *P*
	Mean ± SD	Mean ± SD	
**Pre-test**	48.42 ± 5.39	48.85 ± 5.63	*t* = −.228
			*P* = 0.820
**Post-test **	59.52 ± 6.52	47.66 ± 4.49	*t* = −4.901
			***P*** ** = .000**
**Test and ** ***P***	*t* = −4.020	*t*= −3.211	
	***P*** ** = .000**	***P*** ** = .001**	

**Table 3.** Intra-Group and Inter-Group Comparison of the Nurses in the Education and Control Groups of the Quality of Nursing Work-Life Questionaire (QNWLQ) Pre-Test-Post-Test Mean Scores (n = 42)

	Experimental group (n = 21)	Control group (n = 21)	Test and *P*
	Mean ± SD	Mean ± SD	
Pre-test	81.00 ± 12.46	82.57 ± 13.91	*t* = −555
			*P* = .579
Post-test	91.08 ± 11.06	79.00 ± 13.16	*t* = −2.846
			***P*** ** = .04**
Test and *P*	*t* = −4.037	*t* = −2.705	
	***P*** ** = .000**	*P* = .007	
